# miR‐30b protects nigrostriatal dopaminergic neurons from MPP(+)‐induced neurotoxicity via SNCA

**DOI:** 10.1002/brb3.1567

**Published:** 2020-03-10

**Authors:** Yu‐fei Shen, Zhuo‐ying Zhu, Shu‐xia Qian, Cong‐ying Xu, Yan‐ping Wang

**Affiliations:** ^1^ Institute of Neurology The Second Affiliated Hospital of Jiaxing University Jiaxing China

**Keywords:** apoptosis, miR‐30b, neurotoxicology, Parkinson's disease

## Abstract

**Objective:**

To explore the function of miR‐30b in pathogenesis of Parkinson's disease (PD) and its underlying molecular mechanism.

**Materials and Methods:**

We used 1‐methyl‐4‐phenyl‐1,2,3,6‐tetrahydropyridine (MPP(+)) as a tool for constructing the PD cell model, using miR‐30b mimics or inhibitors to manipulate miR‐30b level for an experimental model of acquisition. The cell viability of SH‐SY5Y was detected by CCK, and luciferase was used to screen the binding of target genes. The protein levels of SNCA were measured by Western blot. Then, we investigate the changes in pro‐ and anti‐apoptotic markers with or without miR‐30b treatment.

**Results:**

There was a significant low expression of MiR‐30b in MPP(+)‐induced cells. SH‐SY5Y cell viability was rescued by MiR‐30b overexpression. Luciferase experiments showed that MiR‐30b may bind to the 3′‐UTR side of SNCA and inhibited its expression. By Western blot, the SNCA level was markedly decreased by miR‐30b. miR‐30b attenuated the upregulation of Bax and the depletion of Bcl‐2 induced by MPP(+).

## Graphical abstract

miR‐30b SNCA Parkinson's disease.

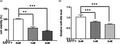

## 1 INTRODUCTION

Parkinson's disease (PD), the second most common neurodegenerative disease in older individuals, usually manifested as a range of activities, including dyskinesia or neurobiological disorders, such as bradykinesia, resting tremor, and muscle stiffness (Abbas, Xu, & Tan, 2018), prominent traits of which include dopaminergic cells loss within the substantia nigra pars compacta (SNpc; Wirdefeldt, Adami, Cole, Trichopoulos, & Mandel, 2011) and the aberrant intracellular protein aggregation including α‐synuclein (Sun, Zhang, Huang, & Chen, 2016). It has been suggested that PD pathogenesis has been implicated to neuronal apoptosis caused by impaired mitochondrial function (Ouazia, Levros, Rassart, & Desrosiers, 2015), so the study of neuronal apoptosis may provide a new target for the treatment of PD (Ghavami et al., 2014). 1‐Methyl‐4‐Phenyl‐pyridinium Iodide (MPP(+)), a commonly used dopamine (DA) neuron toxicant, can cause mitochondrial dysfunction (Nakamura et al., 2000), inducing reactive oxygen species (ROS) pathway activation, eventually leading to cell apoptosis, and is therefore commonly used to simulate PD in vitro model (Suzuki, Mizuno, & Yoshida, 1990).

MicroRNAs (miRNAs) are short, endogenous noncoding RNAs (18–22 nt) that have been associated in countless cell processes in mammals (Ambros, 2003). It has been confirmed that miRNAs play important roles in multiple neural functions (Reinhart et al., 2000). Some enriched or specifically expressed miRNAs in neural system have been suggested to involve with synaptic plasticity, memory, and neuronal differentiation (Kosik, 2006). And there are miRNAs being reported to play a role in neurodegeneration disease (Hebert et al., 2008; Lee et al., 2008; Miska et al., 2004). Moreover, the role of miRNAs has also been suggested in the α‐synuclein aggregation in PD (Kim et al., 2007; Packer, Xing, Harper, Jones, & Davidson, 2008).

In the present study, we analyzed the expression of miR‐30b in MPP(+)‐induced dopaminergic (DA) neuroblastoma cell line SH‐SY5Y. In the gain of function assay, the upregulation of miR‐30b using miR‐30b mimics markedly reduced the death of cells induced by MPP(+). Furthermore, luciferase reporter assay demonstrated that miRNA‐30b could directly bind to the SNCA 3′‐UTR site. Then, we measured the protein or mRNA level of SNCA, and we found that expression of miR‐30b correlated negatively with the expression of SNCA. These findings suggested that miR‐30b could act as a protective factor against neuronal apoptosis induced by MPP(+) via targeting SNCA.

## 2 MATERIALS AND METHODS

### 2.1 Cell culture and treatment

Human neuroblastoma (SH‐SY5Y) and HEK293T cell line was obtained from Cell Bank of Chinese Academy of Sciences. Cells were grown in DMEM medium supplemented with 10% fetal bovine serum, 100 U/ml penicillin G, and 100 μg/ml streptomycin. Cells were maintained at 37°C in a humidified atmosphere with 5% CO2. MPP(+) was added to the cultures to a final concentration of 0.5, 1.0, and 2.0 nM.

### 2.2 MiR‐30b overexpression and inhibition

SH‐SY5Y cells were seeded into 6‐well plates at 2 × 104 per well. After 24‐hr culture, 10nM miR‐30b mimics, miR‐30b inhibitor (Thermo), or miR‐NC was transfected into SH‐SY5Y cells using lipofectamine 2000. After 6‐hr transfection, the medium was replaced by DMEM complete medium.

### 2.3 Luciferase assay

HEK293T cells were seeded into 96‐well plates at 2 × 104 cells per well 1 day before transfection. The SNCA 3′‐UTR mRNA fragment was constructed into psiCHECK luciferase vector (Promega). Then, the established vector and miRNA‐30b or NC were co‐transfected into HEK293T cells using lipofectamine 2000. Then, the activity of luciferase was determined using the Luciferase reporter system (Promega) and measured as the fold change to the basic psiCHECK vector relatively.

### 2.4 RNA isolation and qRT‐PCR

The total RNA of cells was extracted using TRIzol reagent (Invitrogen) following the manufacturer's protocols. The cDNA used to detect gene expression was synthesized with RNA‐to‐cDNA kit (PrimeScript™ RT reagent Kit, TAKARA).

For the determination of the mature miR‐30b, total RNA was reverse‐transcribed by the Mir‐X™ miRNA First‐Strand Synthesis Kit (Clontech) according to the manufacturer's instruction. The small nuclear RNA (U6) served as the internal control. U6 and miR‐30b qRT‐PCR primer are commercially available at Applied Biosystem.

To measure RNA levels, Quantitative TaqManTM real‐time PCR was performed using an Applied Biosystems 7500 fast real‐time PCR system. Primer Express 3.0 (Applied Biosystems) and BLAST (http://blast.ncbi.nlm.nih.gov) were used to design specific primers. Primers used are as follows: hSNCA (fwd primer: AAGAGGGTGTTCTCTATGTAGGC, rev primer: GCTCCTCCAACATTTGTCACTT); hBAX (fwd primer:: TGGCAGCTGACATGTTTTCTGAC, rev primer: TCACCCAACCACCCTGGTCTT); and hBCL2 (fwd primer: TCGCCCTGTGGATGACTGA, rev primer: CAGAGACAGCCAGGAGAAATCA); GAPDH Taqman primers was purchased from Applied Biosystem. All the studies were performed in triplicate. ΔΔ*C*_T_ ($2^{-\Delta \Delta C_{t}}$) method was used for calculation of fold change.

### 2.5 Western blotting

The protein was isolated from SH‐SY5Y cells using RIPA buffer containing protease inhibitor cocktail (Sigma), and aliquots containing 40 μg of total protein were separated into 12% SDS gel and transferred onto 0.45 μm PVDF. After blocking in 5% nonfat milk, the membranes were then incubated overnight with the following primary antibodies: anti‐α‐syn antibody (Abcam) and anti‐β‐actin antibody (Abcam). The secondary antibodies conjugated with horseradish peroxidase were used. The detection of the proteins was carried out by BCA Protein Assay Kit (Thermo) according to the manufacturer's instruction. The proteins were visualized by ECL chemiluminescence and exposed to X‐ray film. All experiments were performed in duplicate and repeated three times.

### 2.6 Cell viability analysis

Cholecystokinin (CCK) assay was performed to measure the cell viability. SH‐SY5Y cells from each group were seeded into 96‐well plates with a density of 1 × 10^4^ per well in 100 μl of culture. To analyze cell viability, 10 μl CCK reagent was added into each well. The plates were incubated at 37°C incubator and subjected to a microplate reader (wavelength: 450 nm).

### 2.7 Statistical analysis

Statistical analyses were performed by the SPSS version 13 (SPSS Inc). All values are presented as the mean ± *SEM* values from different samples from 3 independent preparations. Statistical significance was determined using Student's *t* test. The results were considered significant when *p* < .05.

## 3 RESULTS

**Figure 1:**
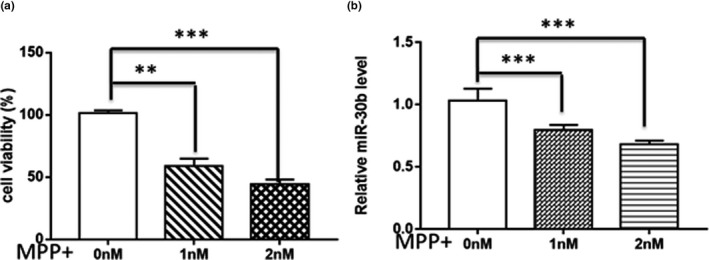
miR‐30b was downregulated in MPP(+)‐treated SH‐SY5Y cells. (a) Analysis of the percentage cell viability in different concentrations of MPP(+). Cell viability of SH‐SY5Y was significantly decreased along with the evaluated MPP(+) concentration. (b) Analysis of miR‐30b expression level in SH‐SY5Y cells treated with 0–2 mM MPP(+). MiR‐30b was detected by qRT‐PCR, and U6 was used as an internal control. Data are presented as the mean ± *SD* of three independent experiments. **p < .05, or ***p < .001

MiR‐30b was significantly downregulated in MPP(+)‐treated SH‐SY5Y cells. We used different concentrations of MPP(+) to find suitable conditions to simulate the PD‐like neuron loss. Cell viability of SH‐SY5Y cells was decreased with the increase in MPP(+) dose (Figure 1a). As shown in the Figure 1b, the expression of miR‐30b in MPP(+)‐treated SH‐SY5Y cells was detected and the level of miR‐30b significantly declined compared to the control group with the change of the concentration (Figure 1b). Based on these results, we selected MPP(+) 2 mm as the likely optimal experimental dose. These results suggested that miR‐30b could affect the toxicity of MPP(+) in SH‐SY5Y cells.

**Figure 2:**
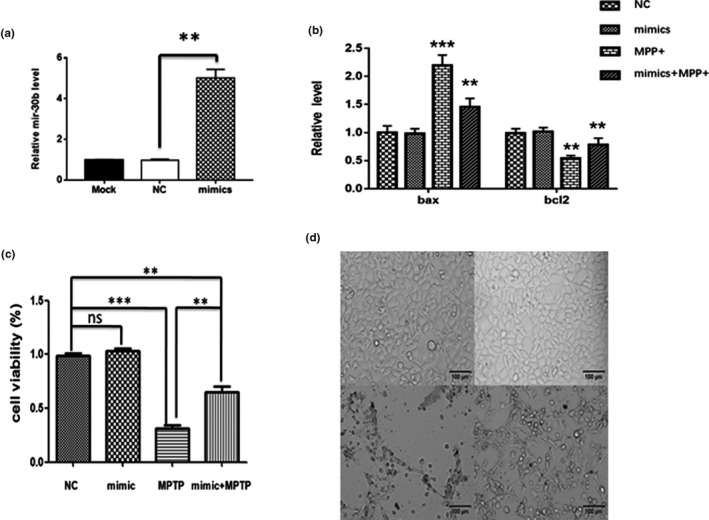
MiR‐30b protected the viability and proliferation of SH‐SY5Y cell from MPP(+). (a) MiRNA level of SH‐SY5Y cells transfected with miR‐30b mimics or NC. (b) Effect of miR‐30b on MPP(+)‐induced Bax and Bcl‐2 expression. (c, d) CCK‐8 assay was performed to determine the viability of SH‐SY5Y cells treating with MPP(+) or not after transfected with miR‐30b mimics (Mimics) or miR‐30b NC, respectively. **p < .05, or ***p < .001

MiR‐30b protected the viability and proliferation of SH‐SY5Y cell from MPP(+). We further transiently overexpressed miR‐30b in SH‐SY5Y cells using miR‐30b mimics to investigate the effects of miR‐30b on cell viability. The expression level of miR‐30b increased about 5.05‐fold compared with the negative control (NC; Figure 2a). Then, we treated the transfected cells with 2 mM MPP(+) and determined cell viability by CCK assay. Compared to the NC group, the cell viability of SH‐SY5Y transfected with mimics was improved in the groups of treating with 2 mM MPP(+) (Figure 2c). Then, we investigate the changes in pro‐ and anti‐apoptotic markers with or without miR‐30b treatment. Cells treated with a MPP(+) manifested significant inductions in Bax and depletion in Bcl‐2. Meanwhile, these alterations were significantly attenuated by co‐treatment with miR‐30b (Figure 2b).

**Figure 3:**
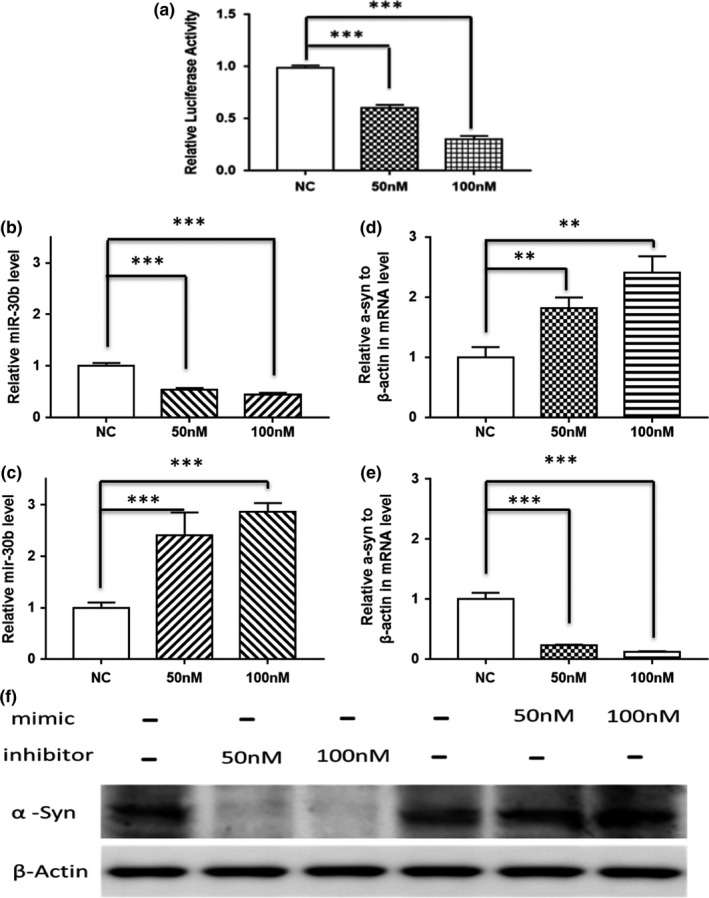
miR‐30b targets the 3′UTR of SNCA and reduces SNCA expression in human neuroblastoma SH‐SY5Y cell line. (a) Luciferase reporter assay was used to determine the binding site. HEK293T cells treated by mimics or NC were transfected with psiCHECK construct containing the SNCA region. (b, c) The manipulation of miR‐30b level in SH‐SY5Y cells. Cells were transfected with 50 or 100 nM miR‐30b mimics and 50 or 100 nM miR‐30b inhibitor or miR control; 24 hr later, the miR‐30b level was examined by RT‐qPCR. (d, e) SNCA expression was suppressed and activated in mRNA level or (f) in protein level in SH‐SY5Y cells. All experiments were performed in triplicate. No significance, **p < .05, or ***p < .001

miR‐30b targets the 3′UTR of SNCA and reduces SNCA expression in SH‐SY5Y cell line. Potential mir‐30b‐targeted genes were predicted and analyzed by bioinformatics analysis (mirDIP, TargetScan); then, the SNCA gene was screened as the candidate gene for miR‐30b (data not shown). As shown in Figure 3a, a luciferase reporter assay was performed to verify whether miR‐30b binds to the 3′ UTR of SNCA. Moreover, we overexpressed or silenced miR‐30b, SNCA mRNA expression was, respectively, reduced or elevated, and the protein level of SNCA was correlated negatively with miR‐30b level (Figure 3f), indicating that miR‐30b negatively regulates the expression of SNCA.

## 4 DISCUSSION

Parkinson's disease (PD), the second most common age‐related neurodegenerative disorders worldwide, has gained more and more attention (Bendor, Logan, & Edwards, 2013). MiRNAs, as a sort of regulatory factors, have been found to play a vital role in the progression of multiple diseases in recent years (Wu & Belasco, 2008). Studies have indicated the important role of microRNAs in neurodegenerative diseases such as Parkinson and Alzheimer's disease (Tan, Yu, & Tan, 2015). For instance, microRNA‐124 could activate brain restoration in Parkinson's disease (Tarazi, Sahli, Wolny, & Mousa, 2014), miRNA‐205 has been reported to regulate the expression of Parkinson's disease‐related leucine‐rich repeat kinase 2 (LRRK2; Cho et al., 2013), and miRNA‐7 protected against MPP(+)‐induced PD cell apoptosis via KLF4, Bax, and Sirt2 (Kong et al., 2016; Li et al., 2016). Furthermore, decreased mir‐34b/34c was observed in multiple associated brain regions (Singh & Sen, 2017). MiRNA‐30b has been reported to be involved in the onset and development of various diseases including glioma cancers and hepatocellular carcinoma. MiR‐30b regulated glioma cell proliferation by directly targeting MTDH (Zhang et al., 2018). In hepatocellular carcinoma, miR‐30b inhibited development of human hepatocellular carcinoma by repressing cell proliferation and cell cycle (Qin, Chen, Wu, & Liu, 2017). In PD, miRNA‐30b may serve as a biomarker. To further verify this, we demonstrated that miRNA‐30b was downregulated in MPP(+)‐induced SH‐SY5Y cells. Next, we overexpressed miRNA‐30b to detect the effect of miRNA‐30b in cell viability and apoptosis in SH‐SY5Y cells. miRNA‐30b may be protective factors against the cell death caused by MPP(+). MiRNAs could affect the stability of translation and inhibit the expression of target genes. We used mirDIP and TargetScan to predict potential target genes for miRNA‐30b and found possible binding sites of mirna‐30b in the 3′‐UTR of SNCA. Alpha‐synuclein (a‐synuclein, SNCA) is a main component of Lewy bodies which is the cardinal pathological hallmark of PD (Braak et al., 2003). Abnormal expression of SNCA was involved in the pathogenesis of both familial and sporadic PD. Some research suggested that aberrant SNCA expression can be related to multiplications of SNCA gene segment. A dosage effect of SNCA duplications and triplication plays a significant role in familial PD (Singleton et al., 2003). In idiopathic PD, studies have indicated that the SCNA gene may harbor significant risk haplotypes (Mizuta et al., 2006). Luciferase reporter assay indicated that miR‐30b could directly bind to the SNCA mRNA 3′‐UTR region. Furthermore, we found that silencing or overexpression of miRNA‐30b significantly increased or decreased the expression of SNCA mRNA and protein levels with qRT‐PCR and Western blot. Moreover, our report also revealed that MPP(+) toxicity results in a reduction of anti‐apoptotic molecule Bcl‐2 and an increase in pro‐apoptotic protein Bax, which were consistent with previous studies (Blum et al., 2001; Wang et al., 2004). Treatment of miRNA‐30b markedly regulated pro‐ and anti‐apoptotic markers. Bcl‐2 played an important role in the maintenance of dopamine neurons in MPTP toxicity (Liu, Chen, Xie, & Wong, 2008). Bcl‐2 also inhibited neuron death by reducing the production of ROS. Bax is a pro‐apoptotic molecule that can be cross‐linked as a homodimer to block the anti‐apoptotic effect of Bcl‐2 (Gross, McDonnell, & Korsmeyer, 1999).Therefore, the effect of miRNA‐30b on MPP(+)‐induced apoptosis may be partially mediated by regulating the expression of Bax and Bcl‐2. All these results indicated that SNCA may serve as a potential target for miRNA‐30b, which reversed the toxic effect of MPP(+) in SH‐SY5Y. Although we have already obtained positive results for miRNA‐30b in MPP(+)‐induced cellular PD model and identify SNCA was an important target of miRNA‐30b, further in vivo studies still needed to illustrate comprehensive mechanism of miR‐30b in PD.

In conclusion, our finding indicated that miR‐30b could play a protective role by inhibiting the apoptosis via suppressing SNCA in MPP(+)‐induced PD cellular model. These data may provide novel insights that have high diagnostic and therapeutic value for Parkinson's disease.

## AUTHOR CONTRIBUTIONS

Yu‐fei Shen designed this study; Yan‐ping Wang supervised the research; Yu‐fei Shen and Zhuo‐ying Zhu performed the experiments; Yu‐fei Shen analyzed the data; Yu‐fei Shen and Zhuo‐ying Zhu wrote the manuscript; and Shu‐xia Qian and Cong‐ying Xu edited the manuscript.

## Data Availability

Data are available on request from the authors.
